# Light regulation of secondary metabolism in fungi

**DOI:** 10.1186/s13036-023-00374-4

**Published:** 2023-08-31

**Authors:** Wenbin Yu, Rongqiang Pei, Yufei Zhang, Yayi Tu, Bin He

**Affiliations:** Jiangxi Key Laboratory of Bioprocess Engineering, College of Life Sciences, Jiangxi Science & Technology Normal University, Nanchang, 330013 Jiangxi China

**Keywords:** Light regulation, Secondary metabolites, VeA, LaeA

## Abstract

Fungi have evolved unique metabolic regulation mechanisms for adapting to the changing environments. One of the key features of fungal adaptation is the production of secondary metabolites (SMs), which are essential for survival and beneficial to the organism. Many of these SMs are produced in response to the environmental cues, such as light. In all fungal species studied, the Velvet complex transcription factor VeA is a central player of the light regulatory network. In addition to growth and development, the intensity and wavelength of light affects the formation of a broad range of secondary metabolites. Recent studies, mainly on species of the genus *Aspergillus*, revealed that the dimer of VeA-VelB and LaeA does not only regulate gene expression in response to light, but can also be involved in regulating production of SMs. Furthermore, the complexes have a wide regulatory effect on different types of secondary metabolites. In this review, we discussed the role of light in the regulation of fungal secondary metabolism. In addition, we reviewed the photoreceptors, transcription factors, and signaling pathways that are involved in light-dependent regulation of secondary metabolism. The effects of transcription factors on the production of secondary metabolites, as well as the potential applications of light regulation for the production of pharmaceuticals and other products were discussed. Finally, we provided an overview of the current research in this field and suggested potential areas for future research.

## Introduction

Secondary metabolites (SMs) are organic compounds that are produced by fungi and other organisms. These compounds are not essential for the survival of the organism, but they can play important roles in various biological processes [1]. For example, SMs can act as signaling molecules to protect the organism from predators or aiding in the production of energy [2]. Besides, SMs can also be used in the production of pharmaceuticals, food additives, and other products (e.g., cordycepin, a major bioactive compound found in *Cordyceps militaris*) [3].

The efficient regulation of secondary metabolism is essential for the survival and growth of organisms in a changing environment. In general, the synthetic gene clusters encoding SMs are in silence [4], and these gene clusters can be activated by various environmental cues, such as light, temperature, and pH [5]. The combination of some techniques can be used to manipulate the production of SMs [6], such as mutagenesisn [7], high-throughput sequencing and bioinformatics techniques, and metabolic engineering [8]. Combining mutagenesis techniques with screening strategies, such as high-throughput assays or genome mining, can help identify mutants with improved SM production [9]. Coexpression analysis reveals tailored fungal metabolic regulation to meet secondary metabolite demands, aiding species selection and engineering high-yield fungal cell factories for production [10]. Synthetic biology tools, such as gene clusters assembly and optimization platforms, enable the construction of artificial pathways for SM production. These techniques offer the potential to create entirely new SMs or tailor existing pathways for improved production [11]. Rational engineering strategies based on insights from genomic and biochemical studies allow targeted modifications to improve precursor availability, increase pathway flux, or remove metabolic bottlenecks. This approach can facilitate the overproduction of specific SMs or the engineering of novel compounds [12]*.* This integrated approach allows for a deeper understanding of the genetic basis and regulation of SM biosynthesis, facilitates the discovery of new compounds, and provides avenues for optimizing yield and expanding the repertoire of bioactive molecules. Moreover, these techniques contribute to the sustainable production of valuable SMs, promoting their industrial and pharmaceutical applications.

The impact of light regulation on fungal secondary metabolism has been demonstrated in various fungal species. For example, in the genus Penicillium, light can regulate the synthesis of penicillin and other polyketide antibiotics [13]. In the yeast *Xanthophyllomyces dendrorhous*, light can induce astaxanthin synthesis [14]. These examples highlight the significant regulatory role of light signals in fungal secondary metabolism. Through the analysis of light-responsive genes and regulatory factors, researchers have unveiled the molecular mechanisms underlying light regulation of fungal secondary metabolism. These findings contribute to our understanding of how fungi utilize light signals to synthesize bioactive compounds and provide potential resources for the development of new drugs and industrial applications. In summary, light signals play a vital role in fungal secondary metabolism. By sensing and regulating gene expression, fungi can produce bioactive compounds. A deeper understanding of light regulation in fungal secondary metabolism opens up new opportunities for the development of fungal-based drugs and industrial applications.

## Regulation of secondary metabolite by light availability

Fungi are sensitive to changes in light quality and intensity due to artificial light sources [15]. The alteration of natural light conditions can disrupt the normal functioning of fungal light-dependent regulatory pathways and secondary metabolite production. Research on the effects of artificial light on fungi and the development of mitigation strategies are areas of active investigation [16]. Some fungi, such as the ascomycete *Neurospora crassa* (*N. crassa*) and the zygomycete *Phycomyces blakesleeanus* (*P. blakesleeanus*), stand out as models for photobiology research [17]. Fungi possess all major classes of photoreceptors, including blue-light receptors, red/far-red light receptors, and phytochromes [18]. These photoreceptors enable the fungi to sense and respond to changes in the environment [19]. In particular, the effects of blue light on fungi have been extensively studied. For example, when *Rhodococcus erythropolis* (*R. erythropolis*) was subjected to blue light (470, 455, 425 nm) exposure, there was a noticeable shift in the fatty acid profiles towards more saturated fatty acids (from C16:1 to C16:0) [20]. Additionally, some fungi have evolved specialized light-sensing structures, such as the sclerotia of *Aspergillus nidulans* (*A. nidulans*), which enable them to sense and respond to light even in low light conditions [21].

Of all environmental signals, the intensity, duration, and quality of light have a special effect not only on development and behavior of fungi, but also on the biosynthesis of many known fungal SMs. Examples include the concentration of carotenoids in *Rhodotorula mucilaginosa* [22], the content of lycopersici, α-tomatine [23], carotenoid [24, 25], fumonisin B_1_ [26] and fusarin in *Fusarium* [27], as well as paracelsin A, paracelsin B and trichodimerol in *Trichoderma reesei* (*T. reesei*) [28]. Additionally, the production of β-carotene in zygomycete fungi biology, such as *Phycomyces blakesleeanus*, *Mucor circinelloides* and *Pilobolus crystallinus*, can be activated by controlling the specific growth rate [29]. Fungi can recognize light signals to evaluate optimal conditions for spore dispersal and activate defense mechanisms against UV light radiation [30]. For example, melanin act as sunscreens to protect against UV radiation in *Paecilomyces variotii* [31], and exposure to blue light can stimulate the production of this pigment. Recent advancements have explored using specific light wavelengths and intensities to enhance the synthesis of desired secondary metabolites with pharmaceutical, agricultural, and industrial significance. These developments offer potential avenues for optimizing fungal secondary metabolite production in controlled environments.

Furthermore, light affects the synthesis of cell wall components through the production of SMs [32]. In particular, exposure to blue light with wavelengths of 470, 455, and 425 nm has been shown to cause a shift in fatty acid profiles towards more saturated fatty acids in *R. erythropolis*, such as C16:0*.* This shift in fatty acid profiles is probably due to the activation of enzymes involved in fatty acid synthesis [32]. Recent research has revealed intricate connections between fungal secondary metabolite production, light perception, and microbiome dynamics. Ballhorn et al. (2016) reported that under light limited conditions, vegetative and reproductive traits were inhibited in arbuscular mycorrhizal fungi (AMF) inoculated *Phaseolus lunatus* plants relative to non-colonized plants [33]. Understanding these complex interrelationships can shed light on ecological processes and potentially lead to the development of sustainable agricultural practices and disease management strategies.

Fungi have different requirements for light intensity, duration, and wavelength, and changes in light availability can trigger various physiological and biochemical responses in fungi [34]. Manipulating light conditions in agricultural settings, storage facilities, or indoor environments can potentially be used as a tool to manage fungal populations [35]. For example, optimizing light spectra or intensities may help favor beneficial fungi or suppress harmful ones, reducing disease incidence or improving plant health [36]. It has been found that *Penicillium* grows faster and produces five times higher ochratoxin A (OTA) under constant light conditions, as compared to growth in constant dark or in alternating light/dark conditions [37]. Thus, in controlled conditions, light intensity and quality (e.g., blue/red ratio) should be modulated to guarantee the symbiosis of SMs [38]. Some reports clearly indicated that light of the same wavelength can either induce or inhibit SMs biosynthesis in different species of fungi [39]. For instance, the exposure of *Penicillium verrucosum* and *Penicillium expansum* (*P. expansum*) to red and blue light has been known to stimulate high biosynthesis of mycotoxin, citrinin [40]. On the other hand, the same light conditions inhibited OTA biosynthesis in *Aspergillus* and *Penicillium* species [41]. However, the potential molecular mechanism of light regulation remains unclear [18]. In particular, the roles of photoreceptors and their downstream signaling pathways in the production of SMs are largely unknown [42]. Red light has been found to promote SMs production in *Monascus*, such as gamma-aminobutyric acid, red pigments, monacolin K and citrinin, while blue light enhances only gamma minobutyric acid production [43]. However, citrinin (CIT) biosynthesis by *Monascus* species decreases in blue light culture conditions and the catalase activity of mycelium can be inhibited by blue light [44]. It was found that both red and blue light influenced pigment yield as well as CIT production of *Monascus* in solid-state or liquid-state fermentation [45]. Similarly, OTA production under red and blue light is greatly inhibited compared to dark hatching, reduced by an average of about 40 times in *Aspergillus niger (A. niger)*. Conversely, red and blue light increase fumonisin B2 (FB2) biosynthesis in *A. niger* [46]. While the CIT biosynthetic gene cluster was found to be up-regulated in *Monascus* [47], the overall effects of blue light on SM production in *Monascus* appear to be largely inhibitory. For example, light can induce the *P. blakesleeanus* to produce carotenoids by four genes, *crgA-D*, which bear similarity to *crgA* in *Mucor circinelloides* (*M. circinelloides*)*.* CrgA is a gene that encodes a repressor of light-induced carotenogenesis [48]. Under blue light conditions fermentation of *Monascus* to synthesize sycamycin, the yield can be increased from 478 mg/L to 689 mg/L [47]. It is possible that the controversial results of the effect of light on fungi may be due to the different types of light sources.

Overall, the regulation of fungal metabolism by light is a complex and multifaceted process that involves the activation of numerous signaling pathways the modulation of gene expression and the enzyme activity. Understanding the specific light-dependent regulators that affect secondary metabolite production in fungi is crucial for manipulating and optimizing their production in various applications, such as pharmaceuticals, food production, and biotechnology. However, it's important to note that the regulation of secondary metabolism in fungi is complex and can vary greatly among different species and their specific secondary metabolite pathways. Therefore, conducting species-specific studies and optimizing light conditions accordingly are necessary to maximize the desired secondary metabolite production in fungi. An overview of fungal SMs affected by light availability is given in Table 1.

**Table 1:** Light-regulated secondary metabolites and regulators involved

Secondary metabolite	Fungus	Light regulation	Light regulators involved	Applications	Reference
Alternariol	*Alternaria alternata*	Light induction	White-collar 1 (WC-1) gene (lreA)	Evaluation and monitoring in food safety and toxicology studies	[49]
Altertoxin	*Alternaria alternata*	Light repression	White-collar 1 (WC-1) gene (lreA)	Ealuation and monitoring in food safety and toxicology studies	[49]
β-carotene	*Phycomyces blakesleeanus*, *Mucor circinelloides* and *Pilobolus crystallinus*	Light induction	Enzymes phytoene desaturase (carB), and the bifunctional phytoene synthase/carotene cyclase (carRA in Phycomyces, carRP in Mucor)	Providing color, increasing nutritional value, and acting as an antioxidant and photoprotectant	[29]
α-tomatine	*Fusarium oxysporum*	Light induction	Photolyase gene (phr1)	Plant protection and medical research	[23]
Lycopersici	*Fusarium oxysporum*	Light induction	Photolyase gene (phr1)	Tomato farming industry	[23]
Carotenoid	*Fusarium fujikuroi*	Light induction	Regulatory gene carS	Providing color, increasing nutritional value, and acting as an antioxidant and photoprotectant	[25]
	*Phycomyces blakesleeanus*	Light induction	Light-sensing MadA-MadB complex		[26]
	*Neurospora crassa*	Light induction	Regulatory protein VE-1, White Collar protein WcoA		[50]
	*Rhodotorula mucilaginosa*	1700 lx	No data		[22]
	*Sordaria fimicola*	Light induction	White collar-1 photoreceptor (SfWC-1)		[51]
Fumonisin B_1_	*Fusarium proliferatum*	green (∼530 nm); yellow (∼590 nm); red (∼627 nm); and blue (∼470 nm)	No data	Evaluation and monitoring in food safety and toxicology research	[26]
Fusarin	*Fusarium fujikuroiis*	Light repression	White Collar protein WcoA	Evaluation and monitoring in food safety and toxicology research	[27]
Sterigmatocystin	*Aspergillus nidulans*	Light repression	Regulator VeA1	Evaluation and monitoring in food safety and toxicology studies	[52]
Paracelsin A, paracelsin B and trichodimerol	*Trichoderma reesei*	Light induction	Transcription factor SUB1	Plant protection	[28]

## Light regulation in fungi: the transcription factors VeA and LaeA

**Fig. 1:**
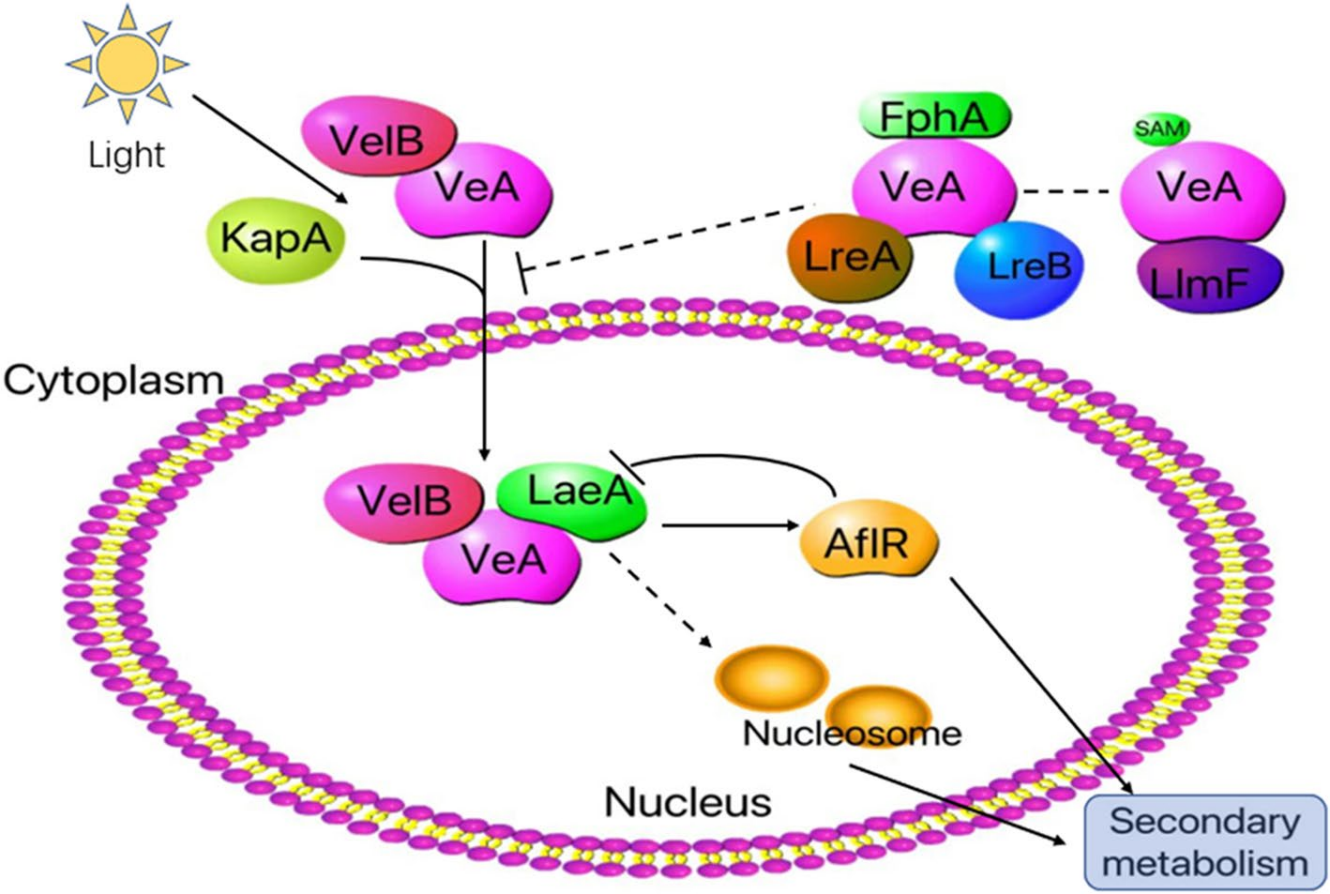
Model of the light regulation of secondary metabolism in *Aspergillus nidulans*. The subcellular localization of VeA in fungi is regulated by various factors. A cytoplasmic VeA-VelB dimer is recognized by importin alpha (KapA) and imported through the nuclear pore complex. Once inside the nucleus, a heterotrimeric complex consisting of LaeA, VeA, and VelB activates secondary metabolism. A transient complex between LlmF and VeA plays a role in repressing the nuclear import of VeA, primarily through the putative methylation activity of LlmF [60]. Furthermore, the red light-sensing phytochrome, FphA, also influences the subcellular localization of VeA. However, it remains unknown whether FphA and LlmF share a common pathway or independently regulate VeA’s subcellular localization

As is widely recognized, the Velvet complex interacts with other regulators and modifies gene expression and is proposed to regulate transcription by DNA binding to promoter regions and chromatin modification [53]. It was initially discovered by Käfer (1965) in *A. nidulans* and comprises of three proteins, namely VeA and VelB (Velvet proteins) and LaeA (Fig. 1), which have been found to positively regulate developmental processes in several *Aspergillus* species [54]. The striatin-interacting phosphatase and kinase (STRIPAK) complex is required for proper expression of the heterotrimeric VeA-VelB-LaeA complex [55]. The discovery that both LaeA and the light-regulated developmental factor VeA are part of the nuclear complex suggests a link between SM production and morphological differentiation [56]. Velvet proteins are composed of two domains, namely the velvet domain and transactivation domain, with the former being unique to filamentous fungi [57]. These proteins can be categorized as either transcriptional activators or repressors based on the type of transactivation domain they possess [58]. The velvet domain is a 150-amino acid domain involved in dimer formation with a structure that resembles the DNA-binding fold of the mammalian transcription factor Nuclear factor-kappa B (NF-κB) [59].

Sequence analysis reveals, that the LaeA gene contains a motif that binds to S-adenosylmethionine (SAM), and the strain after point mutation of the SAM gene exhibits the same phenotype as the strain after deleting LaeA gene. Bayram et al. (2008) reported that LaeA itself has no nuclear localization signal (NLS) [56], and the transcription factors VeA and VelB form a dimer, which is mainly distributed in the cytoplasm in response to illumination. Without illumination, the α-importin protein KapA carries the dimer into the nucleus, where it binds to the nuclear protein LaeA through the SAM site (Fig. 1), forming the Velvet heterotrimer complex and then regulates the production and phenotypic differentiation of SMs [61]. It has been demonstrated that the synthesis of VeA is reduced and the content of LaeA, VeA and VelB heterodimers is diminished under light conditions, consequently inhibiting the synthesis of SMs [62]. These pathways highlight the diverse ways in which fungi respond to and utilize light signals to regulate their growth, development, and physiological processes. The schematic representation helps depict the complex interactions between light sensors, signaling pathways, and downstream cellular responses in fungi, providing a better understanding of the significance of light regulation in fungal biology.

VeA is hypothesized to act as a scaffold protein that mediates developmental pathways in response to illumination by regulating its subcellular localization [5]. Kim et al. (2002) indicated the C-terminal region of VeA contains a putative Pro-Glu-Ser-Thr (PEST) domain. PEST domains are known to be involved in the regulation of protein stability and degradation in eukaryotic cells. Additionally, the VeA protein contains a highly conserved N-terminal domain that is found across various fungal genera [63]. The VeA1 mutant protein lacking the first 36 amino acids at the N-terminus was observed to be mainly present in the cytoplasm regardless of illumination. This suggests that the truncated bipartite NLS in VeA1 is not active or react to light [52], which could clarify why strains carrying the *veA1* allele do not display a light-dependent morphological response. Thus, the subcellular location of VeA has the potential to control protein interaction partners and to direct developmental and chemical responses to environmental cues (Table 2).

**Table 2:** Functions of regulators involved in light-dependent control of secondary metabolism

Regulator	Function	Reference
VE-1	A key component of the velvet complex; and is required for light-dependent transcription; adapt quickly to changes in light exposure by promoting the accumulation of VE-1 for the regulation of genes that participate in the biosynthesis of photoprotective pigments.	[64]
VeA	VeA interacts with other regulatory proteins, such as VelB and LaeA, to form a trimeric complex that controls the expression of secondary metabolite biosynthetic genes, in the dark VeA is located mainly in the nuclei, under light VeA is found abundantly in the cytoplasm.	[52]
VelB	Regulates the expression of several genes involved in the germination process, including the genes for the proteins involved in cell wall degradation.	[65]
VosA	Regulates the expression of several genes involved in the germination process, including the genes for the proteins involved in cell wall degradation	[65]
SUB1	SUB1 not only exerts negative regulation on secondary metabolism as shown during sexual development, but also positively influences metabolite production on minimal medium.	[28]
MadA and MadB	Regulate the mycelial and other asexual processes.	[18]
WCC	The WCC activates downstream signaling pathways that control the expression of secondary metabolite biosynthetic genes.	[66]
LaeA	Interacts with other regulatory proteins, such as VeA and VelB, to control the expression of secondary metabolite biosynthetic genes. controls VeA modification and protein levels and possesses additional developmental functions.	[67]
CarS	A protein down-regulates many genes whose expression is up-regulated by light in wild strains, expression and activation of carotenogenesis by stress, participate in the regulation of genes with catalase domains.	[25, 68]
VosA	Regulates the expression of several genes involved in the germination process, including the genes for the proteins involved in cell wall degradation.	[65]
LreA	Repressing function of some SMs.	[69]

Transcriptional microarray analyses of *laeA* deletion mutants have uncovered the pleiotropic nature of LaeA [70]. The expression of numerous genes and molecules involved in virulence and nutrient acquisition was found to be mis-regulated compared to wild-type strains [71]. Among these genes, *nosA* encodes a Zn (II)_2_Cys_6_ transcription factor important in sexual development in *A. nidulans* and significantly up-regulated in *Aspergillus fumigatus* (*A. Fumigatus*) Δ*laeA* [72]. It contributes to a germination defect of Δ*laeA* mutants [73]. Furthermore, studies suggest that LaeA is also involved in regulating changes in chromatin structure, as the loss of LaeA resulted in increased levels of heterochromatin marks [74].

## The role of the factors VeA and LaeA in regulating secondary metabolism

Secondary metabolism is correlated processes in fungi that respond to light. The veA1 mutant, has been found to exhibit a reduction in nonsporogenous hyphae and an increase in conidia production compared to wild-type strains [63]. Sexual development is also generally delayed and reduced in *veA1* mutant strains, while asexual development is promoted and increased [75]. Based on these findings, the *veA* gene is considered as a negative regulator of asexual development, and its function can be inhibited by light. In 2003, Kato et al. found that the expression of sterigmatocystin and penicillin genes in *A. nidulans* is controlled by VeA [76]. In* A. nidulans*, it has been shown that VeA is a bridge protein between secondary metabolism and light (Fig. 1), whose expression increases during sexual development [77].

It is well-established that *brlA* generates two overlapped transcripts, referred to as *brlA α* and *brlA β*. The expression of *brlA α* is contingent on the presence of both *abaA* and *brlA*, while the overexpression of *brlA β* can stimulate the expression of *brlA α* even in an *abaA* mutant [78]. The *veA* gene is essential for the expression of the transcription factor *aflR* (Fig. 1), which activates the gene cluster that lead to the production of sterigmatocystin in *Aspergillus nidulans* [76]. As mentioned above, the disruption of *veA* affects the expression of a large number of genes in *P. expansum*, including those are involved in secondary metabolism [79]. The *veA* gene and its orthologues have been identified in various fungal species, including *N. crassa* [80] and *Acremonium chrysogenum* [81]. More recently, researchers have discovered that the inactivation of the VeA gene by *Agrobacterium tumefaciens* mediated transformation resulted in a transformant that was still able to produce OTA but in reduced amounts [82]. In *F. verticillioides*, deletion mutants of the VeA orthologue (FvVE1) have been found to repress the production of fumonisins and fusarins [83]. Both VeA and FvVE1 play a role in regulating secondary metabolism [84]. These findings suggest that *veA* orthologues play a conserved role in the regulation of fungal development and secondary metabolism across various fungal species. Furthermore, the discovery of *veA*'s role in regulating mycotoxins production highlights the potential use of *veA* as a target for the development of antifungal therapies to control the production of harmful mycotoxins. Further research is needed to fully understand the molecular mechanisms underlying the function of *veA* and its orthologues in fungal biology.

Studies have shown that deletion of any of the LaeA/VeA/VelB complex can lead to a significant reduction of mycotoxin [85]. For example, in *P. expansum*, the disruption of the *veA* gene drastically reduces the production of patulin and citrinin on synthetic media, associated with a marked down‐regulation of all genes involved in the biosynthesis of the two mycotoxins [79]. Chang et al. showed that LaeA deletion inhibited aflatoxin synthesis, and genes expression involved in early synthesis of aflatoxin, while there was no significant change in transcription of genes involved in late synthesis (*ver1* and *omtA*) [86]. Together, VeA and LaeA act as a switch to control gene expression in response to light. In *Aspergillus ochraceus*, deletion of *laeA*, *veA*, and *velB* result in a 90–95% reduction in OTA production compared to the wild-type strain. Wang et al. (2019) indicated that the deletion of *veA* or *laeA* significantly affected the expression of the backbone genes in OTA gene cluster, among which four out of five were downregulated [87]. The deletion of *laeA* and *veA* genes can reduce the toxin production of *Alternaria alternata* in wheat, thus reduce economic losses when applied to the food industry [88]. Importantly, the genes *veA* and *laeA* played distinct roles in the biosynthesis of SMs in *P. microspora*. For instance, the production of pestalotiollide B, a previously characterized polyketide, required both *velB* and *laeA*. In contrast, the *veA* gene appeared to inhibit the pestalotiollide B (PB) role in its biosynthesis [89].

LaeA is found to interact with various transcription factors, such as NosA, which is negatively regulated by LaeA [73]. Deletion of *laeA* not only reduced the production of several SMs, including the immunotoxin gliotoxin, but also suppressed the expression of 13 SM biosynthetic gene clusters (e.g., *A. fumigatus*-specific mycotoxin clusters). Transcriptomic profiling analysis of WT, Δ*laeA* strains showed that LaeA positively regulates the expression of up to 40% of major classes of SM biosynthetic genes, such as nonribosomal peptide synthetases, polyketide synthases, and P450 monooxygenases [90]. Recent studies have shown that LaeA can also regulate the “cross-talk” between different secondary metabolic pathways, affecting the synthesis of metabolites. For example, in *Penicillium chrysogenum*, the yield of PR toxin in penicillin synthesis is extremely low, and penicillin biosynthesis is inhibited after LaeA gene deletion, while toxin production is increased [91]. In the reverse genetics screen, the new interaction partner of VeA, one of the putative LaeA-like methyltransferases, LlmF (Fig. 1), is a negative regulator of sterigmatocystin production and sexual development in *A. nidulans* [92]. Furthermore, LlmF binds directly to VeA, resulting in increased nuclear accumulation of VeA when LlmF is deleted. This eventually results in decreased expression of VeA-regulated genes. The methyltransferase domain of LlmF is required for its function; however, LlmF does not directly methylate VeA in vitro [60]. The fluP gene in A. flavus is responsible for coding the polyketide synthase enzyme that is essential for the biosynthesis of secondary metabolites in gene cluster 41. This finding indicates that the expression of fluP is positively regulated by developmental regulators of VeA and VelB [93].

In addition, multiple homologous LaeA proteins with similar structures have been identified in *A. niger* [94], *T. reesei* [95] and *Fusarium oxysporum* [96]. Recent research has demonstrated that the LaeA ortholog in *T. reesei*, called LAE1, regulates the expression of cellulases and polysaccharide hydrolases [95]. Mtr23B is a methyltransferase that similar to LaeA, and it shares regulatory functions with LaeA. In particular, Mtr23B has contrasting roles in controlling the formation of conidium pigments and the expression of secondary metabolic gene clusters and glycoside hydrolase genes [92]. MtrA, an ortholog of LaeA in the insect pathogenic fungus *Beauveria bassiana*, is known to have a significant role in virulence and is linked to the control of production of cuticle-degrading enzymes [97]. *T. reesei* Lae1, another ortholog of LaeA, is required for the expression of at least 50 glycoside hydrolase genes [98]. The deletion of *laeA* in *A. flavus* resulted in the loss of aflatoxin production, which is mediated by the loss of expression of *aflR* [99].

## Other regulators involved in light regulation of secondary metabolic processes in fungi

In addition to the Velvet complex, other light regulators are also involved in the regulation of secondary metabolism in fungi (Table 2). These include photoreceptors, such as such as cryptochromes and the White Collar proteins (WCC) for blue light, opsins for green light (retinal-binding proteins) [100], phytochromes for red light [42]. They are involved in the regulation of gene expression in response to light. Other light regulators, such as the circadian clock, are also involved in the regulation of secondary metabolism, as well as in the regulation of the activity of transcription factors [101]. The initial member of this complex was VelA [102]. In the absence of light, VelA forms a heterotrimeric complex with VelB and the global regulator of secondary metabolism, LaeA [103]. Another velvet protein, VosA, also interacts with VelB in the dark [104], and it is suggested that the VosA-VelB heterodimer represses asexual spore formation and regulates spore maturation and trehalose biosynthesis [105]. Under dark conditions, the VelB-VeA dimer in the cytoplasm is transported to the nucleus via interacting with the α-importin protein KapA, where it binds to the nuclear protein LaeA through the SAM site (Table 2), forming the LaeA-VeA-VelB heterotrimeric and promoting the synthesis of SMs and asexual reproduction [67].

Other regulators, such as hormones and small molecules, can also be involved in the regulation of metabolic processes in fungi. VosA and VelB are two NF-kB-like fungal regulators found in *A. nidulans* [65]. In *A. nidulans*, deletion of *velB* significantly reduces the production of sterigmatocystin under light conditions (Fig. 1). In *A. flavus*, deletion of *velB* leads to a significant decrease in aflatoxin production, regardless of whether it is under light or dark conditions [106]. It is possible that the dual role of VelB is to both coordinate with FluG to modulate sclerotial production, as well as to interact with VeA and LaeA to modulate conidiation and aflatoxin biosynthesis of *A. Flavus* [107]. Previous studies have found that the VosA-VelB-WetA signaling pathway is an important regulator of multiple developmental and metabolic processes in *A. nidulans* [108]. Studies have demonstrated that the VosA-VelB complex regulates the expression of several genes involved in the germination process, including the genes for the proteins involved in cell wall degradation [65].

VeA is also known to interact with several other proteins, including the red light sensing protein FphA [109], as well as the blue light sensing proteins LreA and LreB, which are orthologues of the blue-light responsive factors White-Collar1 and White-Collar2 found in *A. nidulans* [49]. FphA serves as a boundary between VeA, LreA, and LreB (Fig. 1), and interacts directly with these proteins [110]. Studies on the effect of light on secondary metabolism in *A. alternata* have revealed that light exposure inhibits the biosynthesis of alternariol and alternariol monomethylether [69]. Pruss et al. (2014) observed that in the *lreA* mutant, ATX (alternariol and alternariol monomethylether) formation was significantly increased in the dark, indicating a repressing function of LreA. However, even in the absence of LreA, *A. alternata* was still able to respond to blue light, indicating the presence of another blue-light receptor system [49].

In 2014, a study discovered a novel regulatory mechanism in *A. nidulans*, in which VapA-VipC-VapB participates in the regulation of growth, development, and secondary metabolism through VeA. Both VipC and VapB contain SAM domains, and VipC shares 52% homology with LaeA [111]. The VapA-VipC-VapB trimer is localized on the cell membrane, and upon stimulation by external light signals, VapA dissociates, and the VipC-VapB dimer enters the nucleus. Nadia et al. (2022) suggested that VelB plays an important role in the regulation of secondary metabolism in *P. expansum* by using a *velB*-deleted mutant strain [112]. The deletion of the *velB* gene in *A. nidulans* (Fig. 1) leads to the enhanced accumulation of brown pigment [113]. On the contrary, in *Fusarium graminearum*, *velB* deletion disrupts pigment synthesis and leads to a reduction in the expression of pigment synthesis-associated genes PKS12, Gip1, and Gip2 [114]. These works provide information for further understanding the biosynthesis of secondary metabolism in the fungus.

Linden et al. (1997) thoroughly studied blue-light sensing responses in *N. crassa* and reported that Zn-finger protein WC-1 and WC-2 forms WCC [115], which on light exposure binds to the promoters of light inducible genes to activate their transcription [66]. In *N. crassa*, the transcription factor complex WCC is essential for most of the light-mediated processes [116]. The WCC temporarily binds to promoters to initiate transcription [117]. The transcription factor WCC is directly activated by light, which resets the clock. Photoadaptation in *Neurospora* is dependent on the blue light receptor Vivid (VVD), which accumulates immediately after light activation and rapidly silences the expression of WCC‐dependent genes [118]. WC-1 is a flavin-binding protein presumed to act as the light sensor. It also has a zinc finger DNA-binding domain and together with WC-2 (also a zinc finger DNA-binding protein) this complex can induce transcriptional changes in response to light [119]. Ballario et al. (1996) found both proteins contain a PAS domain, that mediates their interaction to form a heterodimer known as WC complex [120], and a DNA-binding zinc-finger domain. Liu et al. (2003) indicated that the WCC complex is transiently activated by light and binds regulatory elements of light-regulated target genes to activate their transcription [121]. Further, Estrada & Avalos (2008) described *wcoA* gene in *F. fujikuroi*, which could be a homologue of white-collar protein of *N. crassa*, and this *wcoA* gene was involved in the regulation of secondary metabolism and conidiation in *F. fujikuroi* and had no role in photocarotenogenesis [27].

The Velvet complex coordinates fungal development, secondary metabolism, and light responses by regulating gene expression of the three components of the Velvet complex (VE-1, VE-2, and LAE-1), such as *N. crassa* [50]. VE-1 is barely observed in aerial hyphae in the dark, however, the presence of light is a signal that reduces the degradation of VE-1 so that the protein can be quickly translocated to the nucleus to interact with the the transcription factor genes *vib-1* and *fl* to regulate transcription [122]. The accumulation of VE-1 during the early stage of asexual development requires light exposure [64]. Both VE-1 and VE-2 are equally important for the repression of conidiation and for the activation of carotenoid biosynthesis in *N. crassa* by light. Three velvet proteins VE-1, VE-2, VOS-1, and a putative methyltransferase LAE-1 show light-independent nucleocytoplasmic localization. The expression of VE-1 and VE-2 in *A. nidulans* can successfully replace the light-dependent carotenoid biosynthesis functions of VeA and VelB by forming two functional chimeric velvet complexes in vivo [50]. Gil-Sánchez et al. (2022) have shown that this light effect requires the blue-light photoreceptor WC-1, so they proposed that this new effect of light allows the fungal cell to adapt quickly to changes in light exposure by promoting the accumulation of VE-1 for the regulation of genes that participate in the biosynthesis of photoprotective pigments [64].

## Light availability affects the histone modification patterns

Epigenetic regulation is a regulatory mechanism based on nucleic acid modifications, and the interplay between histone modifications co-regulate gene expression, ultimately affecting SM synthesis in fungi [123]. Light can influence the expression of genes through the modification of histones, such as acetylation, methylation, and phosphorylation. Among these, histone methylation and acetylation are the most extensively studided [124]. Methylation is involved in various biological processes, such as fungal development, circadian rhythm regulation, expression of SM gene clusters, synthesis of hydrolytic enzymes, and formation of pathogenic fungal virulence [125]. In fungi, histone methylation modifications are co-regulated by histone methyltransferases (HMTs) and histone demethylases (HDMs). HMTs can be divided into two categories based on the targeted amino acid residues: lysine (K) methyltransferases (KMTs) and protein arginine (R) methyltransferases (PRMTs) [126]. Light can affect fungal DNA methylation levels, which may be related to LaeA, which has methyltransferase activity. The relationship between fungal light response and these two regulatory factors still need further research [127]. The function of LaeA is consistently linked to its potential to control epigenetic modifications, which may be attributed to its putative protein methyltransferase activity, particularly towards histone tails, as evidenced by the presence of SAM motifs [128]. In the nucleus VeA interacts with VelB and LaeA [87], which has been suggested as an epigenetic regulator for its methyltransferase functions toward amino acid lysine and arginine [129]. Yu et al. (2020) identified a global regulator and named the protein RlcA (Regulator of Light and Chromatin remodeling Activity), which appears to associate with chromatin structure modification. RlcA is localized in the nucleus and interacts with the photosensitive pigment FphA. Its PHD-finger domain may be involved in binding to trimethylated lysine 4 on histone H3 [130].

Histone acetylation is regulated by histone acetyltransferases (HATs) and histone deacetylases (HDACs). Histone acetylation and deacetylation are like switches that can control gene expression and silencing. Histone acetylation can affect the biosynthesis of fungal SMs. The study by Osorio-concepción et al. found that the histone deacetylase HDA-2 of Trichoderma atroviride was induced by light [131]. These results indicate that light can regulate fungal secondary metabolism through epigenetic regulation. Taking the induction of carotenoid synthesis by light as an example, the *crgA* gene is a key negative in light-induced carotenoid biosynthesis in filamentous fungi [132], and it is regulated by light. The expression product of *crgA* has ubiquitin ligase activity, which regulates carotenoid synthesis by catalyzing the ubiquitination of other proteins involved in the carotenoid synthesis process [133]. Therefore, it is speculated that under the influence of light, pathway-specific transcription factor gene expression is regulated, which in turn regulates the expression of relevant SM gene clusters.

Genes involved in the biosynthesis of SMs are often found in clusters. This arrangement allows for co-regulation of gene expression, which can lead to increased production of the metabolites [134]. Some clusters of SM genes are silenced by heterochromatic histone marks, which are mediated at the level of histones by the conserved activator of SM, the LaeA [103]. However, the specific substrate that LaeA directly methylates has yet to be identified, although it has been demonstrated to be self-methylated at Met 207 [135]. Studies have revealed that LaeA is capable of controlling a specific region with well-defined borders that covers around 70 kb of the sterigmatocystin cluster in *A. nidulans* [136]. As a result, LaeA is thought to counteract H3K9 methylation in the sterigmatocystin gene cluster [137]. The crucial functions of the putative histone methyltransferase LaeA in the biosynthesis of SMs and cellulolytic enzymes have been well established [138].

## Conclusion

The ability of fungi to perceive extracellular and intracellular light is essential for their survival and growth. By sensing and responding to changes in light levels, fungi can adjust their behavior and gene expression to optimize their chances of survival in their environment. It is important to understand the effects of different wavelengths of light on fungal metabolism and the production of SMs. By carefully controlling the light conditions under which fungi are grown, researchers can potentially manipulate SM production to promote the production of useful compounds while inhibiting the production of harmful toxins.

Light is a crucial environmental cue for fungi, and its impact on secondary metabolism has been extensively studied in various species. Light can exert its influence directly on photoreceptors or indirectly via the activation of signaling pathways. Photoreceptors, such as phytochromes and cryptochromes, sense light and transduce the signal into changes in gene expression, which result in the upregulation or downregulation of SM production. Furthermore, light also modulate the activity of transcription factors or enzymes involved in secondary metabolism, consequently regulating gene expression and facilitating the production of SMs. Light also regulate the timing of secondary metabolism and the biosynthesis of distinct types of SMs, such as antimicrobial compounds or antioxidants. Lastly, light can be utilized to manage the production of SMs in industrial settings, enabling the efficient production of desired compounds, such as pharmaceuticals [139].

The control of fungal metabolism in response to light is a complex and multifarious process, which entails the activation of multiple signaling pathways, modulation of gene expression and enzyme activity. A deep comprehension of the impact of light on fungi can aid researchers in devising novel strategies to manage fungal growth and alleviate the detrimental impacts of fungal pathogens. The Velvet complex, a crucial component in coordinating fungal development and adapting to diverse light conditions, is responsible for regulating a series of processes such as phototropism, photomorphogenesis, and the biosynthesis of secondary metabolites. Bayram conducted extensive research on the regulatory mechanism of Velvet complex in fungi and demonstrated that light regulates the secondary metabolism and growth differentiation of fungi, the VelB/VeA/LaeA complex plays a crucial role in this regulation. Recent studies have revealed that the ultraviolet receptor CryA can also affect the expression of VeA. These findings indicate that the VelB/VeA/LaeA protein complex synergistically regulates fungal development and secondary metabolism through the formation of complex polymers with its own proteins and other photoreceptor proteins.

## Data Availability

Not applicable.
